# Development of a quadruple qRT-PCR assay for simultaneous identification of highly and low pathogenic H7N9 avian influenza viruses and characterization against oseltamivir resistance

**DOI:** 10.1186/s12879-018-3302-7

**Published:** 2018-08-15

**Authors:** Yang Yang, Shanqin Li, Gary Wong, Sufang Ma, Zhixiang Xu, Xiaonan Zhao, Hong Li, Wen Xu, Haixia Zheng, Jingyan Lin, Qi Zhao, Wenjun Liu, Yingxia Liu, George F. Gao, Yuhai Bi

**Affiliations:** 1 Shenzhen Key Laboratory of Pathogen and Immunity, State Key Discipline of Infectious Disease, Second Hospital Affiliated to Southern University of Science and Technology, Shenzhen Third People’s Hospital, Shenzhen, 518112 China; 2 CAS Key Laboratory of Pathogenic Microbiology and Immunology, Collaborative Innovation Center for Diagnosis and Treatment of Infectious Disease, Institute of Microbiology, Center for Influenza Research and Early-warning (CASCIRE), Chinese Academy of Sciences, Beijing, 100101 China; 3 Yunnan Center for Disease Control and Prevention, Kunming, 650022 China; 4 National Institute for Viral Disease Control and Prevention, Chinese Center for Disease Control and Prevention (China CDC), Beijing, 102206 China; 5 University of Chinese Academy of Sciences Medical School, Chinese Academy of Sciences, Beijing, 101408 China

**Keywords:** Highly/low pathogenic avian influenza virus, H7N9, Quadruple qRT-PCR, Molecular diagnostics, NAI-resistance, Oseltamivir

## Abstract

**Background:**

During the fifth wave of human H7N9 infections, a novel highly pathogenic (HP) H7N9 variant emerged with an insertion of multiple basic amino acids in the HA cleavage site. Moreover, a neuraminidase inhibitor (NAI) resistance (R292K in NA) mutation was found in H7N9 isolates from humans, poultry and the environment. In this study, we set out to develop and validate a multiplex quantitative reverse transcript polymerase chain reaction (qRT-PCR) to simultaneously detect the presence of H7N9 and further identify the HP and NAI-resistance mutations.

**Methods:**

A quadruple qRT-PCR to simultaneously detect the presence of H7N9 and further identify the HP and NAI-resistance mutations was designed based on the analyses of the HA and NA genes of H7N9. This assay was further tested for specificity and sensitivity, and validated using clinical samples.

**Results:**

The assay was highly specific and able to detect low pathogenic (LP)- or HP-H7N9 with/without the NAI-resistance mutation. The detection limit of the assay was determined to be 50 genome-equivalent copies and 2.8 × 10^− 3^ 50% tissue culture infectious doses (TCID_50_) of live H7N9 per reaction. Clinical validation was confirmed by commercial kits and Sanger sequencing with ten clinical samples.

**Conclusions:**

We developed and validated a rapid, single-reaction, one-step, quadruple real-time qRT-PCR to simultaneously detect the presence of H7N9 and further identify the HP- and NAI-resistance strains with excellent performance in specificity and sensitivity. This assay could be used to monitor the evolution of H7N9 viruses in the laboratory, field and the clinic for early-warning and the prevention of H7N9 infections.

## Background

Since human H7N9 infection was first identified in 2013 [1], mainland China has experienced five waves of human infections [2, 3]. H7N9 virus infection caused acute respiratory distress syndrome and resulted in high morbidity and mortality [1, 4]. As of March 27, 2018, a total of 1567 cases of laboratory-confirmed H7N9 virus infections were identified with a case fatality rate of ~ 39% (615 deaths) [5]. During the first four waves, the H7N9 viruses circulating in China were classified as low pathogenic (LP) avian influenza virus (AIV) carrying amino acid residues PKGR/G at the HA cleavage site, causing asymptomatic infection in poultry [4, 6]. However, a highly pathogenic (HP) H7N9 AIV variant emerged during the fifth wave in 2017, and has since been identified in 28 human infections [7–10]. These HP-H7N9 isolates contained polybasic amino acids with a PKGKRTAR/G, PKGKRIAR/G, PKRKRAAR/G or PKRKRTAR/G in the cleavage site of HA proteins [2], which are associated with enhanced virulence in chickens [2, 11].

Antivirals play an important role in the management of influenza virus infections [12]. Neuraminidase inhibitors (NAIs) have been used as front-line therapeutic options since the novel H7N9 viruses contain the S31N mutation in the M2 protein, which confers resistance to the M2 ion channel blockers such as amantadine [1, 13]. However, studies have shown that the NA R292K (N2 numbering) mutation in H3N2 and H7N9 viruses [13–16], H274Y (N2 numbering) mutation in the seasonal H1N1, H1N1pdm09 and H5N1 viruses [17–21], emerged during the treatments or through spontaneous changes [14, 22, 23], resulted in a high level of resistance to oseltamivir and other NAIs. Furthermore, H7N9 viruses with the NA R292K mutation were frequently reported in the infected patients, and were usually accompanied by prolonged virus shedding and adverse clinical outcomes [7, 16, 24]. The continuous emergence of NAI-resistant viruses increases the potential threat of the H7N9 viruses to public [25].

Sensitive molecular techniques are needed for the rapid detection of HP-H7N9 variants and NAI-resistance to monitor its circulation, transmission and guide antiviral treatments in clinic. Currently, two qRT-PCR assays have been reported to identify HP-H7 or NAI-resistance mutation of H7N9 virus [26, 27]. However, the application may be restricted by the limit of detection and the circumscribed detection targets. To this end, we developed and validated a multiplex qRT-PCR to simultaneously detect H7N9 and further identify the HP and NAI-resistance strains, and the results are presented herein.

## Methods

### Viruses and RNA extraction

The strains of influenza A and B were previously isolated, and preserved by our group. Viral stocks were prepared in embryonated chicken eggs (Xinxing Dahuanong Biotechnology, Guangzhou, China) with the approval of Animal Ethics Committees from Shenzhen Third People’s Hospital. Viral RNA was extracted from stocks using the QIAamp RNA Viral Kit (Qiagen, Heiden, Germany) according to the manufacturer’s instruction. Viral RNA was eluted in 50 μL of RNA-free buffer and stored at − 80 °C for subsequent use.

### Viral stock titration by TCID_50_

MDCK cells (ATCC, Manassas, USA) in 96-well plates were grown to 90% confluence and infected with 10-fold serial dilutions of the H7N9 chicken embryo allantoic fluid for 1 h at 37 °C. Then the cells were overlaid with fresh DMEM plus TPCK-treated trypsin (2 μg/ml). At 3 days post infection (dpi), plates were assessed for the lowest dilution in which 50% of the wells exhibited cytopathology and HA titer. The 50% tissue culture infectious dose (TCID_50_) values were calculated according to the Reed-Muench method [28].

### Primer and probe design

All the HA and NA sequences of H7N9 used in the present study were downloaded from the NCBI (National Center for Biotechnology Information) and GISAID (Global Initiative on Sharing All Influenza Data), and aligned using the MEGA 7.0 software. Regions covering the insertion mutation in HA cleavage site and the NAI-resistance mutation (R292K) in NA were chosen as the targets for the primer design. The primers and probes were designed using the Primer Premier 5 software. The probes were labeled with four different fluorescent reporter dyes including FAM, VIC, CY5, and ROX at the 5′-end. The FAM and VIC were labeled with the fluorescent quencher dye NFQ-MGB at the 3′-end, while CY5 and ROX were labeled with BHQ. The sequences and genome positions of the primer and probe set are shown in Table 1.

**Table 1:** Primers and probes used in the multiplex qRT-PCR assay

Primers/probes	Sequences (5′-3′)^a^	Genome positions^b^	Final concentration (nM)
H7-F	GGRAAATGYCCRAGATATGTTAA	934–956	600
H7-R	AATTAGKCCTTCCCATCCATTT	1062–1083	600
H7-P-W	ROX-TTGGTGCYATAGCDGGTTTCATTGA-BHQ2	1037–1061	600
H7-P-M	FAM-AGRGAAAACGGRYTGCGA-MGB	1010–1027	800
N9-F	GAAGAATGCTCATGTTACGG	832–851	400
N9-R	TGACTAGTGTGTGTCATTGCTA	929–950	400
N9-P-W	Cy5-ATTGGCAGGGCTCAAATAGACCAGT-BHQ3	887–911	400
N9-P-M	VIC-GCACATGCAAGGACA-MGB	872–886	400

^a^FAM, 6-carboxyfluorescein; Cy5, cyanine 5; ROX, 6-carboxy-X-rhodamine; MGB, minor groove binding; BHQ, Black Hole Quencher; Y = T or C, R = A or G, K = G or T, D = A or T or G

^b^Nucleotide position is based on the ORF of HA and NA genes of A/Anhui/1/2013(H7N9) (GISAID accession: EPI_ISL_138739) except the probe H7-P-M, which is based on the HA gene of A/Guangdong/Th005/2017(H7N9) (GISAID accession: EPI_ISL_250424)

### Generation of DNA standards

A section of the HP-H7 and N9-K292 genes were amplified with the primers H7-F/H7-R and N9-F/N9-R, respectively. The PCR products were purified using the Gene JET PCR Purification Kit (Thermo, MA, USA) and ligated to the pGEM-T vector (Promega, Madison, USA). Plasmids were quantified using the Nanodrop 2000 Spectrophotometer (Thermo, MA, USA). The copy number (molecules/mL) was calculated using the following equation: [C × A / 660 × L], in which C represents the concentration of plasmid (g/mL) assessed by the optical density measurement; A is the Avogadro number (6.023 × 10^23^); L is the length of the plasmid (number of nucleotides); and 660 is an approximation of the molecular weight of a nucleotide (g/mol).

### Quantitative reverse transcription polymerase chain reaction

The samples (both clinical and cultured) were tested by a one-step multiplex qRT-PCR in an ABI QuantStudio Dx Real-Time cycler (Applied Biosystems, Foster City, USA). The One Step PrimeScript™ RT-PCR Kit (Takara, Dalian, China) was used as follows: 0.8 μL enzyme mixture (including reverse transcriptase [RT] and Taq polymerase), 10 μL 2 × One Step RT-PCR buffer III, 0.4 μL of each primer and probe for NA gene, 0.6 μL of each primer for HA gene, 0.6 μL of the universal probe for HA gene (H7-P-W), 0.8 μL of the specific probe for HP-H7 gene (H7-P-M), 0.8 μL RNase free water, and 5 μL RNA (total 20 μL/reaction mixture). The concentration of all the primers and probes was 20 μM. The qRT-PCR assay conditions were as follows: reverse transcription for 5 min at 42 °C; 10 s at 95 °C for reverse transcriptase inactivation and DNA polymerase activation followed by 40 cycles of 5 s at 95 °C and 30 s at 60 °C (annealing-extension step). The data were analyzed using the QuantStudio™ Real-Time PCR Software (Applied Biosystems, Foster City, USA). Commercial qRT-PCR kits for the detection of HP-H7N9 viruses were also used (Mabsky Biotech Co., Ltd., Shenzhen, China; Zhengzhou Zhongdao Biotechnology Co., Ltd., Henan, China) following the manufacturers’ instructions. All samples were analyzed in triplicate with three independent runs.

### Clinical samples

Sputum samples were obtained from ten confirmed cases of H7N9 infection in Shenzhen Third People’s Hospital and Yunnan Center for Disease Control and Prevention. Viral RNA was extracted using the QIAamp RNA Viral Kit (Qiagen, Heiden, Germany) according to the manufacturer’s instruction.

## Results

### Design of the primer-probe set

First, we downloaded and compared the HA genes of the HP- and LP-H7N9 virus. The HA genes of the HP-H7N9 virus possessed either a KRTA, KRIA, or KRAA insertion with a nucleic acid 12 bp in size, 5′-AAA CGG A(G)C(T)T GCG-3′. Thus, we designed the forward (H7-F), reverse (H7-R) primers covering this region, and a probe (H7-P-M) which can specifically recognize the specific insertion. Additionally, a universal probe (H7-P-W) targeting both the HP- and LP-H7N9 within the same region was designed to identify the presence of H7. We then downloaded and compared the NA genes of the H7N9 viruses, selected the forward (N9-F), reverse (N9-R) primers covering the R292K mutation region, and designed a probe (N9-P-M) which can specifically recognize the R292K mutation. Meanwhile, a universal probe (N9-P-W) within the same region was designed to detect the presence of N9 genes. The four probes were labeled with four types of different fluorescence. The oligonucleotide sequences, genome positions, labeled fluorescence of the primers and probes for the multiplex assay are listed in Table 1.

### Specificity of the multiplex qRT-PCR assay

**Fig. 1:**
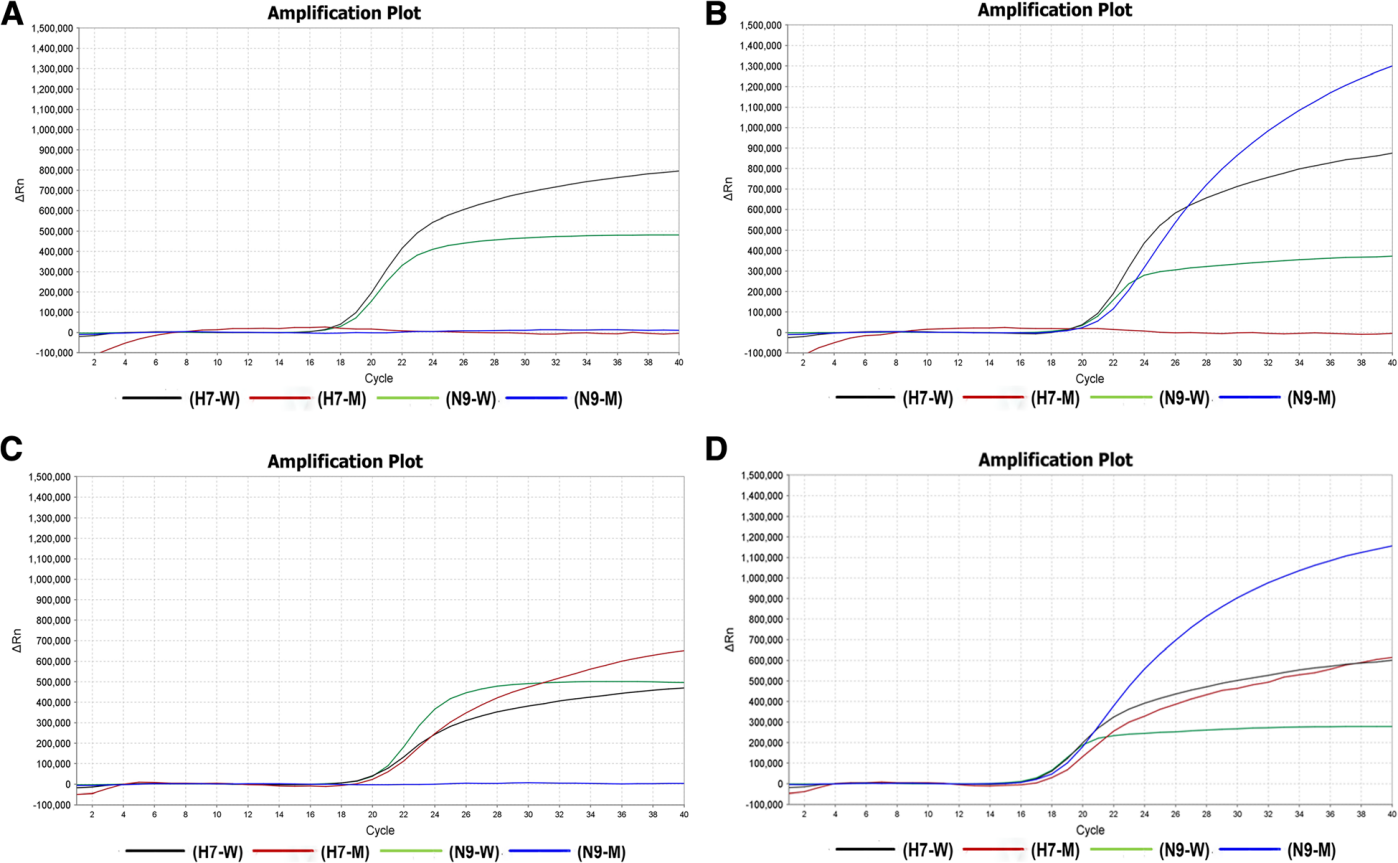
Validation and optimization of the multiplex qRT-PCR assay using different H7N9 viruses. (**a**) Representative amplification plot for viral RNAs from LP-H7N9 without NAI-resistance mutation, (**b**) LP-H7N9 with NAI-resistance mutation, (**c**) HP-H7N9 without NAI-resistance mutation and (**d**) HP-H7N9 with NA inhibitor-resistance mutation. H7-W: detection target for universal H7; H7-M: detection target for highly pathogenic H7; N9-W: detection target for universal N9; N9-M: detection target for R292K mutation of N9

The primer-probe set was optimized as a single-well method for H7N9 detection by one-step multiplex qRT-PCR method as described in the Methods section, and the optimized concentrations of the primers and probes are shown in Table 1. To test the specificity of our multiplex qRT-PCR assay, four different H7N9 viruses (LP-H7N9 without NAI-resistance mutation, LP-H7N9 with NAI-resistance mutation, HP-H7N9 without NAI-resistance mutation and HP-H7N9 with NAI-resistance mutation) were used (the identities of viruses used are listed in Table 2). The universal probes of H7 and N9 successfully detected all four phenotypes of H7N9 virus (Fig. 1). Meanwhile, specific probes targeting HP-H7 or N9-K292 specifically recognized the corresponding phenotypes of H7N9 viruses without any nonspecific amplification. In addition, there were also no nonspecific amplification when tested against other influenza viruses (including H1N1, H3N2, H5N6, H6N6 and H9N2 subtype of influenza A and influenza B) (Table 2).

**Table 2:** The influenza virus strains used in the present study

Types and subtypes	Reference strains	GISAID NO.	HP/LP AIV	292R/K in NA	Virus titer (TCID_50_/mL)	qRT-PCR assays			
						H7-W	H7-M	N9-W	N9-M
H7N9	SZ/Th003	EPI_ISL_250313	LP	R	2 × 10^6^	14.38 ± 0.12	U^c^	14.44 ± 0.21	U
H7N9	SZ/Th004	EPI_ISL_250314	LP	K	2 × 10^6^	14.37 ± 0.36	U	15.05 ± 0.23	14.15 ± 0.55
H7N9	GD/Th005	EPI_ISL_250312	HP	R	2 × 10^6^	15.61 ± 0.48	15.06 ± 0.49	15.45 ± 0.19	U
H7N9	SZ/Th007	EPI_ISL_259757	HP	K	2 × 10^6^	13.91 ± 0.24	13.87 ± 0.56	14.45 ± 0.15	13.55 ± 0.09
H3N2	CAS0001	—^a^	—^b^	—^b^	—^a^	U	U	U	U
H1N1	CA04	FJ966082	—^b^	R	—^a^	U	U	U	U
H5N6	Th001	EPI_ISL_205503	HP	R	—^a^	U	U	U	U
H9N2	04.15SZBAXQ005	EPI_ISL_199448	LP	R	—^a^	U	U	U	U
Flu-B	Victoria	—^a^	—^b^	—^b^	—^a^	U	U	U	U
Flu-B	Yamagata	—^a^	—^b^	—^b^	—^a^	U	U	U	U

^a^not available

^b^not applicable

^c^undetected

### Sensitivity of the multiplex qRT-PCR assay

**Fig. 2:**
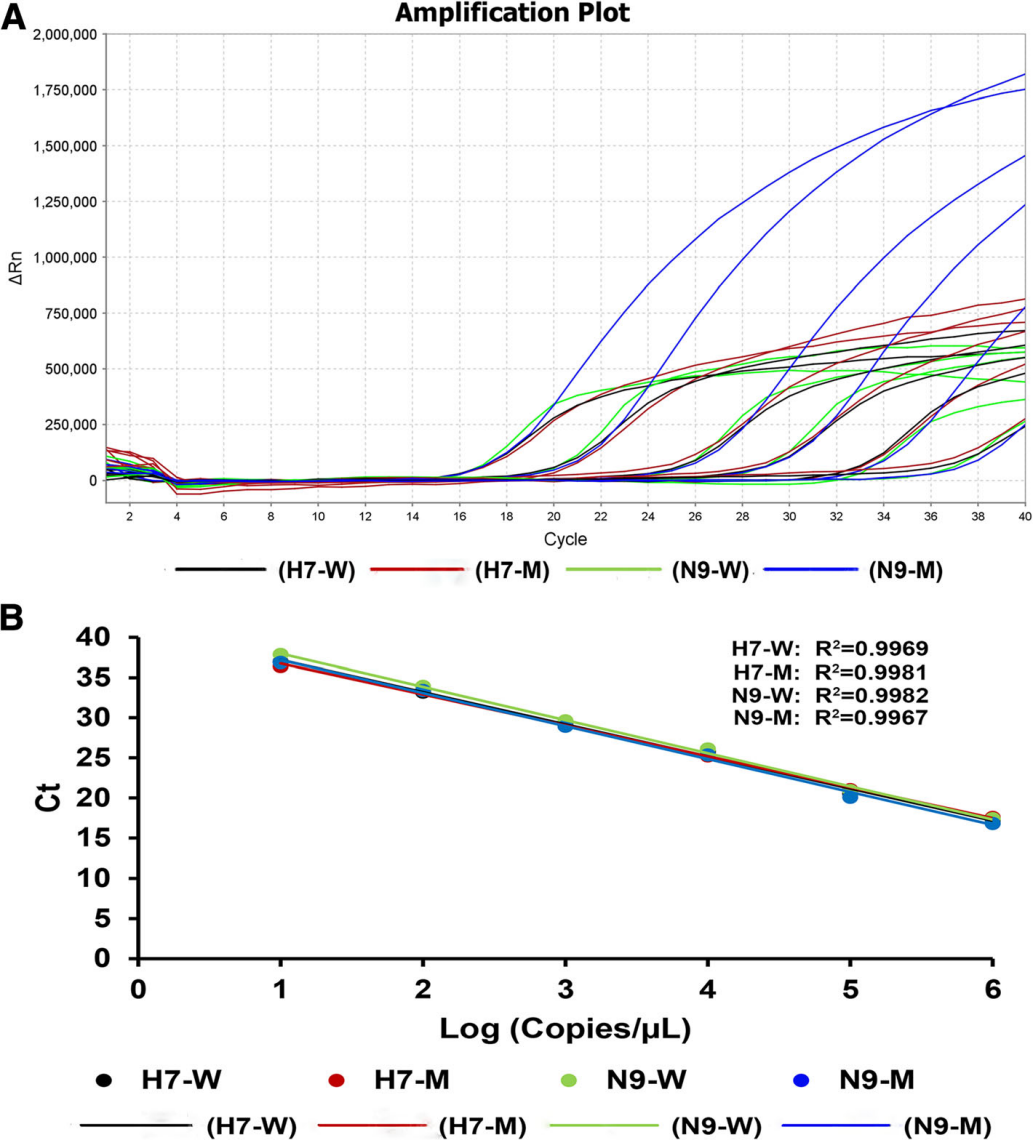
Sensitivity of the multiplex qRT-PCR assay using DNA standards. (**a**) Representative amplification plot of the different concentrations of plasmids expressing the targeted H7 or N9 genes (10-fold dilutions). (**b**) Standard curve for ten-fold serial dilutions of the DNA standards. The log numbers of plasmids (Copies/μL) are expressed linearly on the *x*-axis, whereas Ct values obtained from qRT-PCR are expressed linearly on the *y*-axis. H7-W, H7-M, N9-W and N9-M are defined the same with Fig. 1

The detection limit of the multiplex qRT-PCR assay was evaluated by quantitative standards from a pGEM-T vector expressing the target sequence of the HP-H7 and N9-K292 genes, or viral RNAs prepared from serial ten-fold dilutions of the four different phenotypes of H7N9 viral stocks. The multiplex qRT-PCR method using DNA standards as template showed that Ct values were similar among the four targets, and the linear range was between 1 × 10^6^ to 1 × 10^1^ molecules, respectively. Moreover, the regression coefficients (R^2^) values were above 0.99, indicating that the assay was both accurate and precise over this range (Fig. 2). The limit of detection was determined to be 50 genome-equivalent copies per reaction, based on specific amplification curves (Fig. 2a) and the standard curve (Fig. 2b).

**Fig. 3:**
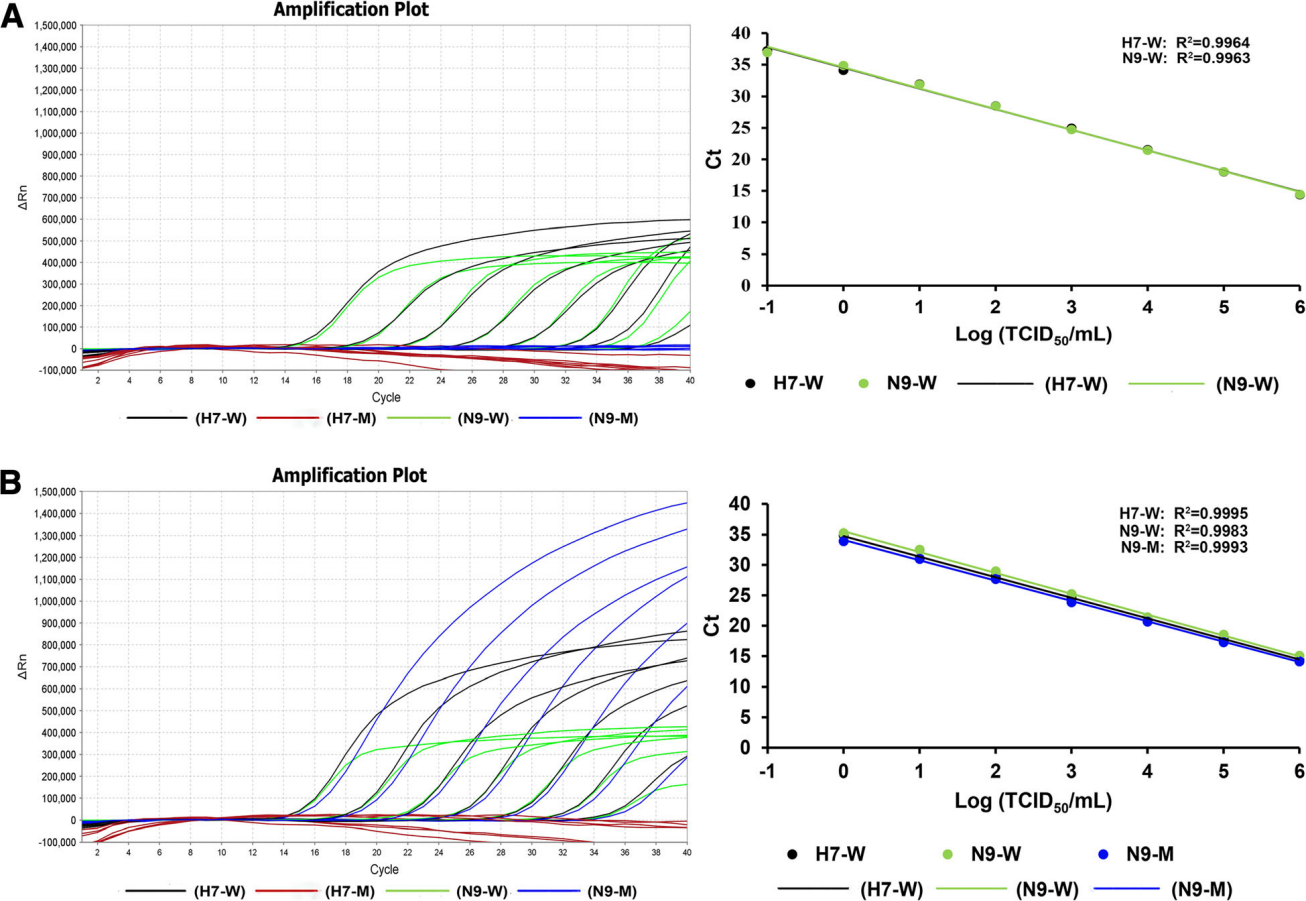
Sensitivity of the multiplex qRT-PCR assay using viral RNAs from LP-H7N9 viruses. (**a**) Representative amplification plot and standard curves for ten-fold serial dilutions of LP-H7N9 without NAI-resistance mutation strain. (**b**) Representative amplification plot and standard curves for ten-fold serial dilutions of LP-H7N9 with NAI-resistance mutation strain. The log numbers of live H7N9 viruses (TCID_50_/mL) are expressed linearly on the *x*-axis, whereas Ct values are expressed linearly on the *y*-axis. H7-W, H7-M, N9-W and N9-M are defined the same with Fig. 1

**Fig. 4:**
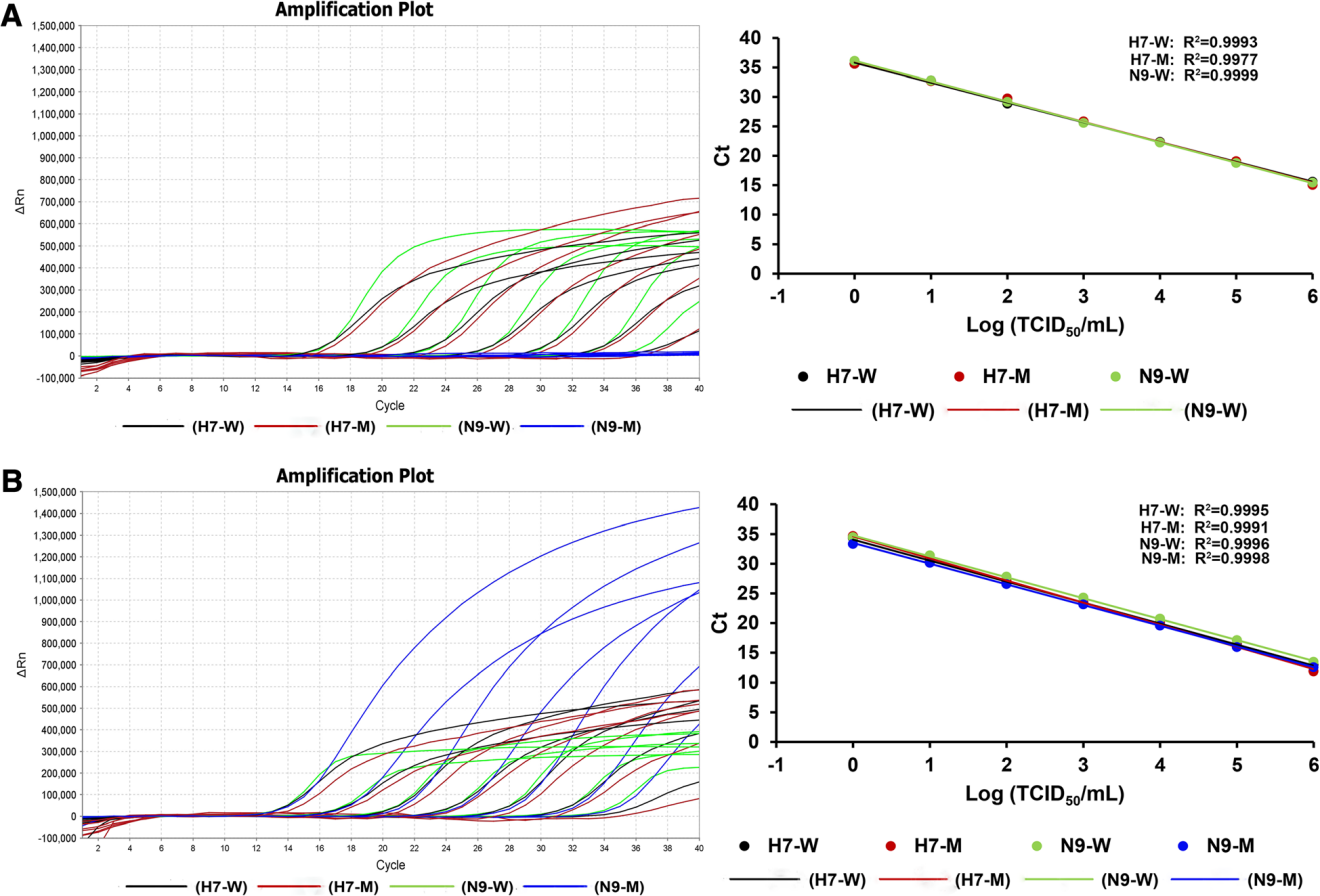
Sensitivity of the multiplex qRT-PCR assay using viral RNAs from HP-H7N9 viruses. (**a**) Representative amplification plot and standard curves for ten-fold serial dilutions of HP-H7N9 without NAI-resistance mutation strain. (**b**) Representative amplification plot and standard curves for ten-fold serial dilutions of HP-H7N9 with NAI-resistance mutation strain. The log numbers of live H7N9 viruses (TCID_50_/mL) are expressed linearly on the *x*-axis, whereas Ct values are expressed linearly on the *y*-axis. H7-W, H7-M, N9-W and N9-M are defined the same with Fig. 1

Meanwhile, RNA samples were extracted from ten-fold serial dilutions of four phenotypes of stock H7N9 viruses ranging from 2 × 10^6^ to 2 × 10^− 2^ TCID_50_/mL, and were used to test the detection limit (Figs. 3 and 4). Results showed that the detection limit of the multiplex qRT-PCR assay was similar among the four H7N9 viruses (2.8 × 10^− 2^ TCID_50_) per reaction, except for LP-H7N9 without NAI-resistance, which was 2.8 × 10^− 3^ TCID_50_ per reaction. The titers correlated well with the obtained Ct values (R^2^ > 0.99) (Figs. 3 and 4).

### Validation of the multiplex qRT-PCR assay in clinical samples

To validate the assay with clinical samples, we tested 10 sputum specimens obtained from 10 patients confirmed to be infected with influenza A (H7N9) virus during the fifth wave (Table 3). As expected, all samples tested positive for H7N9 viruses with similar Ct values compared to two commercial kits (Table 3). Furthermore, samples from patients SZ05, SZ07 and SZ08 were determined to be HP-H7N9; and samples from patients SZ04, SZ07 and SZ08 were determined to have NAI-resistance mutations. These results were confirmed by Sanger sequencing.

**Table 3:** Detection of H7N9 virus in the clinical samples

Clinical samples	H7 (HP/LP)^a^				N9 (292R/K)		GISAID NO.
	Multiplex qRT-PCR	Kit-1	Kit-2	Sanger sequencing	Multiplex qRT-PCR	Sanger sequencing	
SZ01	32.5 (LP)	32.46 (LP)	33.28 (LP)	LP	R	R	EPI_ISL_250424
SZ02	35.92 (LP)	35.85 (LP)	35.52 (LP)	LP	R	R	EPI_ISL_250425
SZ03	31.79 (LP)	31.26 (LP)	31.9 (LP)	LP	R	R	EPI_ISL_250313
SZ04	26.74 (LP)	27.16 (LP)	27.05 (LP)	LP	K	K	EPI_ISL_250314
SZ05	26.04 (HP)	26.74 (HP)	25.28 (HP)	HP	R	R	EPI_ISL_250312
SZ06	26.91 (LP)	26.35 (LP)	26.43 (LP)	LP	R	R	EPI_ISL_250315
SZ07	28.57 (HP)	27.95 (HP)	28.91 (HP)	HP	K	K	EPI_ISL_259757
SZ08	19.81 (HP)	20.29 (HP)	20.22 (HP)	HP	K	K	EPI_ISL_266936
YN01	27.35 (LP)	28.31 (LP)	29.05 (LP)	LP	R	R	EPI_ISL_250316
YN02	36.03 (LP)	35.22 (LP)	35.13 (LP)	LP	R	R	EPI_ISL_250317

^a^The Ct values of the commercial kits and our developed method for universal H7 were shown

## Discussion

Previous studies have revealed that the HP-H7N9 virus has caused several outbreaks in poultry farms, in which over 110,000 poultry were found dead in at least 10 provinces [29]. Meanwhile, human infections in rural areas were mostly associated with exposure to sick and dead poultry [8], and the HP-H7N9 variants were also found in the live poultry markets (LPMs) and environment of the human patients [2, 30, 31]. In terms of this situation, the rapid and accurate detection method of HP-H7N9 strains is in urgent need. Recently, one group has developed a duplex qRT-PCR assay to detect both LP-H7 and HP-H7 genes [26]. However, another two novel insertion mutations with “KRIA” or “KRAA” motif in the cleavage site has been found for the HP-H7N9 [2, 8, 11, 30], their probe targeting HP-H7 could not cover all the mutations of HP-H7N9 strains. Furthermore, besides H7, our assay also included the detection target of N9 gene, which could further confirm the presence of H7N9 virus. Except for the broader coverage of probes for HP-H7 and the additional detection target of N9, our assay also possessed an excellent sensitivity. Together, our assay may represent an upgrade of the detection method for HP-H7N9 viruses.

The emergence of an NAI-resistance mutation is temporally associated with a rebound of virus load, treatment failure and a poor clinical outcome [16]. Therefore, identification of the NAI-mutation in time may increase the cure rate for H7N9 infections. Resistance to NAIs in particular has important implications, as this class of agent is most widely used for treatment and outbreak control. Accordingly, appropriate laboratory methods for the continuous efforts of laboratory surveillance are of great importance [12]. Currently, the gold standard methods for antiviral resistance screening include phenotypic and genotypic methods using NA-Star^®^ assay and Sanger sequencing, respectively [32, 33]. While both of these methodologies are labor intensive, time-consuming and expensive, suggesting the need for a rapid, high-throughput approach to influenza drug resistance testing. Although an SNP real-time RT-PCR for detection of NAI-resistance mutation has been developed [27], the application may be restricted in its delayed detection after the identification of H7N9 and the limit of detection. As described in our assay, we can directly determine the presence of LP- or HP-H7N9 viruses and simultaneously identify the NAI resistance with higher detection limit, which significantly improve the efficiency and performance of detection.

## Conclusions

In conclusion, we developed and validated a rapid, single-reaction, one-step, quadruple real-time qRT-PCR to simultaneously detect the presence of H7N9 and further identify the HP- and NAI-resistance strains with excellent performance in specificity and sensitivity. As the ongoing circulation of H7N9 viruses among poultry, enhanced surveillance is critical to guide prevention and control efforts. This assay will be of great use in monitoring the circulation of HP-H7N9 and NAI-resistant virus strains during AIV surveillance and the treatment of patients with NAIs to prevent persistent viral replication and severe inflammatory reactions.

## Abbreviations

AIV: Avian influenza virus
HP: Highly pathogenic
LP: Low pathogenic
NAI: Neuraminidase inhibitor
qRT-PCR: Quantitative reverse transcript polymerase chain reaction
SNP: Single nucleotide polymorphism
TCID_50_: 50% tissue culture infectious dose
